# Clinical Nurses’ Expectations and Experiences of Self‐Directed Online Training in Evidence‐Based Practice: A Qualitative Study ‐ #EvidencerProject

**DOI:** 10.1155/jonm/5549295

**Published:** 2026-07-03

**Authors:** Antonio Jesús Ramos-Morcillo, Serafín Fernández-Salazar, José Antonio Vera-Pérez, Jessica García-González, María Ruzafa-Martínez

**Affiliations:** ^1^ Department of Nursing, Faculty of Nursing, University of Murcia, Murcia, Spain, um.es; ^2^ Andalusia Care Strategy, Andalusian Health Service, AGS Northeast Jaén, Úbeda, Jaén, Spain, juntadeandalucia.es; ^3^ Department of Nursing, Faculty of Nursing, University of Murcia, Cartagena, Spain, um.es; ^4^ Faculty of Health Sciences, University of Almeria, Almería, Spain, ual.es

## Abstract

**Background:**

Evidence‐based practice (EBP) is essential for improving the quality of care and health outcomes. Despite its importance, nurses’ competence in EBP remains limited, emphasizing the need for more effective training strategies.

**Aim:**

This study aimed to explore the learning expectations and experiences of clinical nurses who took part in the #Evidencer Project, a self‐directed online course on EBP.

**Method:**

A qualitative approach was used, applying reflexive thematic analysis. Participants were experienced clinical (Hospital Care *n* = 10, Primary Care *n* = 5) nurses who had completed a 72‐h free online training course on EBP. The participants had heterogeneous educational backgrounds (generalist nurses, *n* = 4; specialist nurses, *n* = 3; master’s degree, *n* = 6; doctorate, *n* = 2). Semistructured virtual interviews were conducted and analyzed following Braun and Clarke’s method. The MAXQDA 24 software supported the analysis process. Trustworthiness was ensured through a collaborative and reflective coding process carried out by two researchers, an audit trail, triangulation with existing literature, and explicit attention to researcher reflexivity.

**Results:**

A total of 15 nurses participated in the study. Three main themes emerged: (1) motivation and expectations: motivation was primarily intrinsic, driven by personal interest in EBP, the need for professional development, and their roles as student tutors; (2) experience with the training: participants positively evaluated the clarity, flexibility, quality of the content, and nursing‐adapted design, although areas for improvement were identified, such as the need for tutoring and better support for complex content; (3) EBP worldview: a partial understanding of the EBP process was noted, focused on the initial phases (steps 0–2: problem identification and evidence search), with greater difficulty in the advanced phases (steps 3–6: critical appraisal, implementation, evaluation, and dissemination). Added to this was the confusion between research and EBP.

**Conclusion:**

The #Evidencer Project, self‐directed online training in EBP was well regarded for its accessibility, flexibility, and quality, but participants perceived it as insufficient as a standalone strategy for comprehensive EBP implementation. The nurses indicated a need for institutional support for the comprehensive application of EBP, highlighting the importance of training strategies integrated within the clinical context and backed by the institutions.

## 1. Introduction

Evidence‐based practice (EBP) is widely recognized as a foundational approach to improving healthcare quality and patient outcomes [1]. It is currently known that the implementation of EBP leads to better patient outcomes, along with a good return on investment in healthcare systems [2]. This approach is particularly relevant in the current clinical context, where the complexity of healthcare and the need for optimal patient results require a systematic method based on scientific data [3]. In addition, the World Health Organization (WHO) believes that EBP is a priority field of action that enhances the contribution of nurses to the health of citizens [4].

EBP integrates the best available scientific evidence, the clinician’s expertise, and the patient’s values and preferences to guide clinical decision‐making [3]. The EBP process is structured into seven steps: cultivating a spirit of inquiry, formulating clinical questions, searching for the best evidence, critically appraising the evidence, integrating it with clinical expertise and patient preferences, evaluating outcomes, and disseminating results [5].

In recent years, it has been established, as a fundamental requirement, that all health professionals understand and apply EBP principles, considering them as a minimum standard of professional competence [6]. However, before aspiring to a high level of efficiency in implementing clinical interventions, it is important to start from a lower level to consolidate the basic EBP competencies among professionals [7]. At the international level, recent studies have shown that nurses’ proficiency in this field remains limited, with the nurses’ EBP competence generally being low or moderate [7, 8]. As a result, health systems must intensify their efforts to guarantee an adequate EBP training that will improve the competencies of these professionals and promote the practical application of EBP in real‐world clinical settings.

Despite advances in face‐to‐face training, this modality presents limitations of accessibility, scheduling rigidity, and high cost, particularly for practicing professionals. Self‐directed online learning emerges as a theoretically promising alternative grounded in adult learning principles that emphasize autonomy, self‐regulation, and flexible engagement with knowledge [9]. However, to date, online EBP training interventions in nursing have been evaluated predominantly from quantitative perspectives, without exploring in depth the lived experience of professionals.

This study is grounded in Self‐Directed Learning (SDL) theory [10, 11], which posits that adults learn more effectively when they take responsibility for their own learning process, set their own objectives, and select their resources autonomously. This framework informed the design of the interview guide, centered on participants’ goal‐setting, resource selection, and self‐regulatory strategies during training. It also shaped our analytical lens when interpreting differences in engagement and expectations among participants.

The continuing education of nurses has been oriented toward the use of online methodologies, adopting different approaches, such as self‐directed learning, blended learning, and interactive learning [9]. The literature review shows that e‐learning techniques used in nursing have a heterogeneous design and application, including a combination of self‐guided modules, synchronous training, multimedia, simulation, and case studies [12]. E‐learning for nurses presents significant advantages, such as easy access to knowledge, temporal and geographical flexibility, and determinant factors like cost‐effectiveness and internet availability. However, some barriers also exist, such as technological competencies, limited institutional support, lack of motivation, and limited time due to other priorities. Despite these challenges, many systematic reviews suggest that digital education is well‐received and not inferior to traditional education [13, 14], showing that e‐learning improves clinical knowledge and practice, favoring the professional development of nurses [9].

Improving EBP competencies of clinical nurses through digital education interventions can be a very attractive educational alternative. The studies conducted to date, through the application of online training programs, are quantitative in nature and show a positive impact on the acquisition of knowledge, skills, and use of EBP [15–20]. However, these studies have been conducted in very heterogeneous contexts, and the training characteristics applied, such as duration, content, or online teaching modality, are very variable, which makes it challenging to provide a solid set of recommendations [19].

On the other hand, the high variability of the training programs and the need to evaluate their development underline the importance of deeply exploring the significance of receiving online EBP training for nursing professionals. In this sense, qualitative research is an ideal methodology, as it allows us to understand, in detail, the experiences, perceptions, and challenges nurses face during their learning process. This approach facilitates the identification of barriers and facilitators in the implementation of educational strategies, as well as the contribution to theoretical–practical learning, of different learning methods in clinical and academic contexts. In addition, qualitative research helps to analyze how the training received influences decision‐making and professional practice from the perspective of the professionals, thus allowing for the improvement of education programs and their alignment with the real needs of the professionals and healthcare systems [21–23].

To our knowledge, few qualitative studies have explored nurses’ perceptions and learning experiences regarding self‐directed online EBP training. Therefore, the aim of this study is to examine the learning expectations and experiences of clinical nurses who participated in the “#Evidencer Project,” a self‐directed online training program on EBP.

## 2. Methods

### 2.1. Design

A qualitative methodology was employed using a reflexive thematic analysis [24, 25] to address the study objective.

The study was reported according to the Consolidated Criteria for Reporting Qualitative Research (COREQ) [26].

### 2.2. Study Setting and Subjects

The study is part of the #Evidencer Project, a Spain‐based study aimed to evaluate the level of EBP competence among Spanish nurses [27] and to determine the effectiveness of online training in increasing EBP competence [16].

The nurses who participated in the research were recruited from a sample that collaborated in the initial phase of the project and expressed their interest in continuing their participation in the study. The inclusion criteria specified that they worked in the national healthcare system (hospital or primary care setting) and had at least 1 year of clinical experience. In addition, completion of the full #Evidencer online training program was an explicit inclusion criterion: all 15 participants who took part in the interviews had completed the entire course, as the interview questions addressed the full learning experience across all seven modules.

The online EBP training had the following characteristics: it was free, accredited by the University of Murcia, with a workload of 72 h (3 European Credit Transfer and Accumulation System (ECTS)) and a duration of 12 weeks. The training took place on an online platform designed by the researchers (AJRM, MRM), who had extensive experience teaching EBP. It was specifically designed for nurses and intended for the self‐study of the participants, offering materials in various formats (text, video, and web content), clinical scenarios, and interactive activities. The contents were structured considering the seven steps of EBP according to the model proposed by Melnyk et al. [5]. Each step corresponded to a learning module, Table 1. At the end of each module, the participants had to take an assessment test. Each module had to be completed within a defined time, and moving on to the next one depended on the time spent, the performance of the activities, and passing the corresponding test.

**TABLE 1:** Description of the online evidence‐based practice course.

Modules	Hours	Contents
Module 0: Cultivate a spirit of inquiry within an evidence‐based practice (EBP) culture and environment	6	Analysis of the evolution of science and its impact on nursing and medical practice Discussion about the concept of EBP, advantages, and disadvantages Review of clinical variability examples Reflections on barriers, clinical responsivity, and ethics

Module 1: Ask the burning clinical question in PICOT format	6	Importance of asking clinical questions Formulation of clinical questions following the PICOT format Clinical domains of PICOT questions and their relationship with research designs

Module 2: Search for and collect the most relevant best evidence	18	Criteria for conducting a search of scientific evidence. Resources for scientific evidence. How to conduct searches in bibliographic databases of health sciences Analysis and examples of searches for scientific evidence in PubMed

Module 3: Critically appraise the evidence	18	Reflections on why it is important to conduct critical reading of scientific evidence Understanding statistics to comprehend science Critical reading of systematic reviews and clinical practice guidelines

Module 4: Integrate the best evidence with one’s clinical expertise and patient/family preferences	12	Steps to implement practice change in an organization with examples of evidence implementation Importance of leadership for practice change in the clinical context

Module 5: Evaluate outcomes of the practice decision or change based on evidence	6	Sources of internal evidence Measurement of outcomes to evaluate the impact of changes

Module 6: Disseminate the outcomes of the EBP decision or change	6	Reflections on the importance of disseminating the results and achievements obtained Dissemination through various means: posters, oral presentations, and scientific articles

For heterogeneous population groups, a sample size of between 5 and 25 people is recommended for in‐depth/semi‐structured interviews [28].

Maximum variation sampling was operationalized by identifying candidates with heterogeneous profiles across the following criteria: years of professional experience (range: 3–37 years), care setting (hospital vs. primary care), sex (female/male), current professional role (clinical nurse, midwife, supervisor, or union representative; note that in Spain midwifery is a nursing specialization requiring a postgraduate qualification), level of additional training (specialization, master’s, doctorate), type of employment contract (permanent, interim, or temporary), and prior EBP training hours (< 40 h, 40–150 h, > 150 h, or none). Participants were selected from the available sample seeking balance across these dimensions.

The final sample size was not predetermined but was evaluated progressively throughout data collection in accordance with the concept of information power [29]. In keeping with the epistemological foundations of reflexive thematic analysis [24, 25], sample adequacy was assessed by considering the following intersecting dimensions: the specificity of the sample with respect to the research aim; the use of established theory as an analytical lens; the quality and richness of the interview dialogs; and the analytical strategy employed (cross‐case thematic analysis). These considerations informed a provisional sample range, and the final decision to conclude data collection was made in situ, based on the richness, complexity, and analytical sufficiency of the accumulated data to address the research question.

### 2.3. Data Collection

The participants were contacted via email to invite them to participate; none of them refused to participate. They selected the day and time for the interview and agreed to participate in the study. Individual semi‐structured interviews were conducted using an open‐ended question script that included questions related to expectations regarding online EBP training, experiences with the training program, and suggestions for improving the online training. A pilot test of the interview script was conducted earlier with three clinical nurses and the research team.

Two research team members (S.F‐S., J.G‐G.) with more than 10 years of research experience conducted the interviews virtually from January to March 2023. They audio recorded, transcribed verbatim, and analyzed the recordings. Notes were taken after each interview, and codes were used to guarantee the confidentiality of the participants.

### 2.4. Analysis

The six phases proposed for the thematic analysis were followed [30]: (1) Familiarizing yourself with your data; (2) generating initial codes; (3) searching for themes; (4) reviewing themes; (5) defining and naming themes; and (6) producing the report. The recorded interviews were transcribed verbatim. The sample was considered analytically adequate when the data demonstrated sufficient richness, complexity, and depth to generate a coherent and multifaceted thematic description of the phenomenon under study [29, 31]. In line with the epistemological premises of reflexive thematic analysis, the decision to conclude data collection was based on an iterative assessment of informational power: following each interview, the research team reflected on whether the data continued to expand the analytical complexity and depth of the emerging themes, and whether the sample as constituted contained sufficient information to address the research objectives rigorously. This assessment led to a final sample of 15 participants, at which point the data were deemed analytically sufficient. The MAXQDA 24 software was used.

The trustworthiness of the study was enhanced through: (a) a collaborative and reflective coding process carried out by two researchers, in which interpretative discrepancies are resolved through iterative discussion, with consensus understood as a means of enriching analytical depth rather than as a means of ensuring the reliability of the coding [24]; (b) an audit trail documenting analytical decisions; and (c) triangulation of findings with the existing literature.

Regarding reflexivity, it should be noted that the #Evidencer training program was designed by two members of the research team (AJRM, MRM) who, given its self‐directed online format, were not involved in its delivery. The researchers responsible for conducting the interviews and analysis (SFS, JGG) had limited involvement in the course design; both are experienced clinical nurses and educators with established views on EBP, which may have facilitated rapport with participants but also predisposed them to certain interpretations of the data. To minimize the potential influence of both prior project knowledge and researcher positionality on the analytical process, coding discrepancies were resolved by consensus, reflexive notes were maintained throughout, emerging themes were discussed with a third team member (JAVP), and findings were triangulated with the existing literature.

### 2.5. Ethical Aspects

The research was approved by the Research Ethics Committee of the University of Murcia (ID: 2540/2019). Before their participation, the participants were informed about the objectives, what their participation would entail, and their ability to leave the study at any time without explanation. The confidentiality of the data provided was assured, as was their anonymity and the use of anonymized analysis.

## 3. Results

Fifteen nurses participated, with a mean age of 43.5 years and 20 years of professional experience. The majority were women (86.6%)—10 in a hospital setting and 5 in primary care. The detailed composition of the sample is shown in Table 2.

**TABLE 2:** Sociodemographic variables.

Sex	Age	Years of professional experience	Position	Other education	Health care setting	Previous EBP training	Type of contract
Female	32	15	Clinical nurse	Doctorate	Hospital Care	< 40 h	Interim
Male	25	3	Midwife	Specialty	Hospital Care	< 40 h	Temporary
Female	58	37	Clinical nurse	Doctorate	Hospital Care	From 40 to 150 h	Permanent
Female	25	3	Clinical nurse	Nursing	Primary Care	from 40 to 150 h	Temporary
Female	25	3	Clinical nurse	Master’s	Hospital Care	> 150 h	Temporary
Female	57	30	Clinical nurse	Master’s	Primary Care	None	Permanent
Female	57	30	Union representative	Nursing	Primary Care	< 40 h	Permanent
Male	45	23	Supervisor	Nursing	Hospital Care	None	Permanent
Female	53	34	Midwife	Specialty	Hospital Care	from 40 to 150 h	Permanent
Female	41	20	Clinical nurse	Master’s	Hospital Care	from 40 to 150 h	Interim
Female	42	20	Clinical nurse	Nursing	Hospital Care	from 40 to 150 h	Permanent
Female	51	30	Supervisor	Master’s	Primary Care	< 40 h	Permanent
Female	52	27	Clinical nurse	Master’s	Hospital Care	None	Permanent
Female	48	15	Clinical nurse	Specialist	Primary Care	< 40 h	Permanent
Female	42	18	Supervisor	Master’s	Hospital Care	< 40 h	Permanent

Three main themes were identified: (1) motivation and expectations for EBP training; (2) experience with online EBP training; and (3) EBP worldview. The themes, subthemes, codes, and their verbatim transcriptions are detailed in Supporting Table S1.

### 3.1. Theme 1. Motivation and Expectations Toward EBP Training

#### 3.1.1. Intrinsic Motivation and Professional Development

The nurses’ motivation for training was their own interest in EBP. This intrinsic motivation drives them to stay current and enhance their competence in this subject. The nurses expressed a consistent interest in EBP training, whether to stay up‐to‐date, reinforce prior knowledge, or explore specific areas such as critical reading of articles. In addition, some emphasized that repeating training activities helped them consolidate their knowledge more effectively.

*“I do little things every year, right now I don’t need it anymore, but I don’t stop training…the topic of evidence-based nursing is one of the things that most interests me, because I like being up to date.”* (E3: Doctorate)

#### 3.1.2. Role as Student Tutor

Another important factor was the interest and pressure they experienced when trying to adequately train students in their care, including undergraduate and graduate students. Their enthusiasm for EBP training was linked to enhancing the quality of teaching and academic support.

*“We tutor Final Degree Project students, and thus, we want to provide quality.”* (E1: Doctorate)

#### 3.1.3. Complementary Training for Advanced Studies

Lastly, they were motivated to take the course as complementary training to support their higher education. The nurses emphasized that they enrolled in this course to supplement their Master’s or Doctoral studies, seeking to reinforce concepts, clarify previous knowledge, and gain more practical research training.

*“I signed up for the course because I thought it could maybe serve me in the Research Master’s (which I’m currently doing).”* (E2: Specialty)

Regarding their expectations, the nurses conveyed unclear ideas in their statements about what the training would entail. Some initial uncertainty was observed among the participants before they began.

*I had no idea; I didn’t have any expectations because I really didn’t know what I was signing up for*. (E8: Nursing)

In this sense, the nurses had limited or unclear expectations about the course due to a lack of knowledge concerning the content or to previous experience with more basic EBP training. Some assumed it would be similar to other courses, while others were pleasantly surprised to discover a broader and more in‐depth approach.

*“I expected less than what it was…I expected the typical training, with some critical reading, or bibliographical search, which I often find at basic levels.”* (E11: Nursing)

### 3.2. Theme 2. Experience With Online EBP Training of the #Evidencer Project

#### 3.2.1. Perceived Value of the Training

The nurses who were interviewed indicated that the training had “value” due to various aspects. On the one hand, they considered the training specifically dedicated to nurses; one interviewee referred to it as “*dedicated to me.”* In this sense, they positively evaluated the examples specific to nursing, as these facilitated application in practice.

A second aspect is that they viewed the course as “*a gift*,” as it was good quality, clear, and free.

In fourth place, another aspect emerged that highlighted the value of the training: they would recommend it to their peers. This demonstrates the level of satisfaction with the course.

*“I have a friend…and I also told her, sign up for this, it’s really good. It has an exceptional quality.”* (E1: Doctorate)

Lastly, they assessed the course as offering practical, clear training focused on key concepts, promoting learning through concise explanations and visual resources.

*“It gave me the feeling that they wanted us to learn key concepts, because they repeated them, or whatever they saw as being practical.”* (E4: Nursing)

#### 3.2.2. Platform Usability

Another subtheme related to the experience with online training was the usability of the web platform. The nurses indicated that the online platform featured a dynamic and animated design. They emphasized organized blocks, videos, games, infographics, and interactive tools. In addition, they highlighted a clear and concise language, as well as the balance between theory and practice, which made learning more accessible and engaging.

*“That’s why I really liked the platform, because it was very dynamic, because it had videos, infographics…”* (E2: Specialty)

#### 3.2.3. Areas for Improvement

Another emerging subtheme was related to identifying improvements in the training. They identified the possibility of some tutorships or synchronous sessions being in‐person to resolve doubts and increase motivation, as well as a greater presence of the trainers to make the experience more engaging.

*“Well, a session with the tutor so (he/she) can give us an explanation, well, especially for motivation, yes, for motivation.”* (E10: Master’s)

The time commitment and duration of the course varied among the nurses’ responses, although some expressed a need for greater flexibility regarding the duration of the course and prolonged access to the content.

*“Let’s see, I would add more (hours) for the content, but yeah, I would leave it open longer, because the truth is that if you work, in the end you start to leave it, and it’s very hard to find time to sit down and do it.”* (E4: Nursing)

Lastly, they missed the opportunity to have downloadable materials, such as PDFs or summaries, for later consultation to reinforce learning.

*“I would have liked to download some of the materials, because you forget them, and you cannot consult them.”* (E11: Nursing)

### 3.3. Theme 3. Evidence‐Based Practice Worldview

This is an emerging theme that provides context and aids in understanding the results obtained globally.

#### 3.3.1. Complexity of EBP and Daily Application

The nurses interviewed possess a comprehensive understanding of EBP, characterized by significant complexity and a degree of reluctance toward its implementation due to the challenging nature of EBP, coupled with the necessity for daily commitment.

*“Yes, it’s an interesting subject. What happens is that I think of it as very complex…it is complicated to…I think to internalize it into everyday life.”* (E8: Nursing)

However, EBP fosters a critical spirit toward clinical practice. Their interest arises from the need to understand and justify clinical practices, challenging tradition and seeking greater efficacy.

*“‘Above all, it stems from my need to understand why I do things*.*”* (E6: Master’s)

The nurses interviewed indicated that the training is meant to be used, but they did not identify it as intended for the patients, who are the final recipients.

*“They benefit from us doing things better, right? Then, maybe it is not a direct application towards them, but in the end, they benefit from what we do.”* (E8: Nursing)

#### 3.3.2. Confusion Between EBP and Research

In addition, they confuse EBP with research by failing to distinguish between the two as separate entities. This confusion is not merely terminological but reflects a deeper conceptual gap in nurses’ understanding of EBP as a distinct process from research production. Participants frequently described research‐related activities (e.g., initiating a study, writing a protocol) when asked about EBP, suggesting that the distinction between “doing research” and “using evidence” had not been internalized.

*“Because there are also times when we want to start a research project or something on the (hospital) ward, some case that is relevant, then we want to know how we could do it.”* (E13: Master’s)

#### 3.3.3. Individual Versus Collective EBP Practice

Finally, nurses distinguish the individual versus the group. They also identify a dual relationship with EBP: the “self” as an autonomous learner and the “self” as a professional within their clinical context.

*“Yes, yes, right now I’m absolutely alone, the feeling…but the feeling is: stay in your consultation, do what you have to do, but don’t’ mess around.”* (E6: Master’s)

## 4. Discussion

The objective of this study was to evaluate the learning expectations and experiences of clinical nurses who participated in the #Evidencer Project, a self‐directed online training course focused on EBP with a workload of 72 h distributed over 12 weeks. In total, 15 clinical nurses from various professional settings and diverse educational backgrounds were interviewed. The results revealed three main themes: motivation and expectations for EBP training; experiences during the online EBP training of the #Evidencer Project; and finally, EBP worldview.

Regarding motivation, it is essential to interpret our findings with the understanding that the course was entirely voluntary, not part of any institutional training program, or provided by health services as continuing education. This aspect may have influenced the finding that one of the primary reasons for participation was the intrinsic interest in EBP among the nurses who took the course. These results align with previous studies that highlighted nurses’ interest in participating in training activities to enhance their professional knowledge and skills [32], viewing this training as an individual responsibility [33].

Another novel aspect that motivated the nurses to take the course was their role as tutors for undergraduate and graduate students, who act as drivers of change. The students promote continuous improvement in professional practice, urging clinical workers to adopt evidence‐based approaches and achieve better results [34, 35]. This phenomenon could help explain why university health centers offer higher‐quality care.

In contrast to other studies in which continuing education is mainly associated with the practice of care [36], in this case, it was observed that nurses seek to strengthen their EBP competencies as a complement to their Master’s and Doctorate degrees, which suggests an association between advanced education and the integration of evidence‐based practice.

The expectations of the participants regarding the training were limited or undefined. The broad and diverse training offerings create uncertainty in the participants’ expectations, as the programs’ quality tends to be unequal. This variability in the training is not a recent problem, but it is still a challenge [37]. Furthermore, the findings emphasize the need to establish clear criteria for nurses to identify high‐quality EBP training programs, streamlining their selection of relevant professional development opportunities.

Regarding their experience with the course, the nurses indicate that its value lies in its specificity, as it was designed for their professional profile. This aspect gains special importance considering that recent research highlights a scarcity of online training tailored for nurses, with a mono‐disciplinary approach, open format, and mass accessibility [38].

Among the identified learning facilitators were clarity, freedom, quality of content, the possibility of self‐managed time, and no need for a physical space for development. Some characteristics, such as access to the content at any time and the convenience of online learning, were pointed out in previous studies on online learning among nurses in other countries [39]. The nurses who took the course also highlighted the importance of the web platform’s usability, the content’s design, and the tools used to present the content. This aspect is a key factor for the adequate development of e‐learning [40]. Other learning facilitators could include external support, offered by accrediting organizations, which is identified as a way to improve motivation and learning results [41, 42].

These findings are consistent with the predictions of SDL theory [10, 11]: participants who managed their own pace and adapted the training to their schedules reported higher satisfaction and greater perceived learning. Conversely, those who experienced difficulty with advanced EBP steps may have lacked the self‐regulatory skills that SDL assumes as preconditions for effective independent learning.

As improvements, the participants proposed tutorships where they could express their doubts and directly associate with the teaching staff. Previous studies coincide with this and suggest providing sessions or workshops with the teaching staff about topics related to the more complex contents [43], underlining that the support of tutors improves the learning experience of the nurses [39]. They also mentioned that the contents related to step 3, “critical appraisal” must be split into at least two different modules due to its complexity. This observation coincides with previous studies that identified critical reading as one of the most complex EBP stages [44]. In addition, other studies suggest that assigning estimated times to each module can be a valuable strategy to improve time management in online training courses [45].

The EBP worldview emerges as a key element that allows us to understand better some matters related to the reach of the training course.

The inherent complexity of EBP learning is a recurring challenge identified in this and previous studies. Clinical practice is not a static individual activity but is constructed through shared, dynamic, and contextual interactions [46], making it difficult to identify the effects of interventions a posteriori and reinforcing nonevidence‐based habits [47, 48]. Hence, EBP training, though positive and necessary, is insufficient without adaptive, progressive learning strategies and institutional scaffolding.

Nurses in this study showed a partial understanding of the EBP process. When describing EBP, they focused almost exclusively on the early stages: cultivating a questioning attitude (step 0), formulating clinical questions (step 1), and searching for information (step 2). They rarely mentioned the later steps: critically appraising evidence (step 3), implementing changes (steps 4–5), or sharing outcomes (step 6). This pattern suggests a two‐tier EBP profile among clinical nurses: a basic level, centered on independent information‐seeking and self‐directed learning, expressed by participants as the “I” as an autonomous learner, and an advanced level, requiring collaborative implementation and institutional support, experienced as the professional “I” isolated in their clinical context, where EBP feels “distant, costly, and difficult” (E6: Master’s; E3: Doctorate). It is important to note that this distinction did not emerge as an inductively derived category from participants’ discourse, but rather represents an interpretative framework developed by the research team to synthesize observed patterns, drawing on established EBP frameworks [49, 50]. The contrast between individual and collective EBP practice was explicitly present: while some participants described years of experience as “lone wolves” (E3: Doctorate), others identified peer leadership and shared unit initiatives as the conditions that made advanced‐level EBP practice possible (E4: Nursing; E13: Master’s). These observations are consistent with the #EvidencerMUSEBP model, which identifies supportive clinical contexts as a primary determinant of nurses’ EBP use [51], and with implementation science frameworks that situate collective adoption, by professional organizations and institutional programs, as a prerequisite for full EBP integration [47].

Although the qualitative design of the study does not allow for statistical comparisons, an analytical reading of the verbatim excerpts suggests that nurses with higher educational levels tended to express a more articulate understanding of the advanced EBP steps and to identify their knowledge gaps more precisely, while nurses with undergraduate training showed greater uncertainty regarding the critical appraisal and implementation steps. This finding points to the need for differentiated training strategies according to the professional’s profile.

It is also significant that among the motivations for taking the course, we found a fundamental absence of EBP in a specific intervention or area. That is, there was no prior motivation to search for a resolution for any particular clinical intervention framed within their clinical context, and the nurses had not even considered the expectation that the course could lead to changes in their practice, a habitual finding in other online training courses [52]. This lack of instrumental motivation may be due, at least in part, to the difficulty of adapting linear models derived from research to the complexity of clinical practice [46].

Another idea that could partially explain the lack of a unified perspective on the EBP process is the persistent confusion between research and EBP [2, 3, 46]. This confusion is illustrated, for example, by the fact that one motivation for taking the course was its usefulness as a complement to some of the nurses’ Master’s or Doctoral studies. Other authors have indicated that on some occasions, EBP is mentioned without the performance of activities that are truly related to it. EBP has become a trendy term often misused or out of context [2]. The WHO and diverse authors emphasize the need for nurses to clearly understand the fundamental concepts of EBP, as their indistinct use in practice and literature can hinder its correct application. This understanding is key for promoting the commitment to EBP and improving the quality of care and health outcomes [7, 53].

An added aspect to analyze is the overall maturity level of EBP practice. The nurses interviewed indicated that this training will have repercussions on their patients, although it does not truly involve their participation. This aligns with other authors, who pointed out the lack of inclusion of patients’ values and preferences in clinical decision‐making and the current need for strategies to implement and assess meaningful patient participation in EBP [54].

Based on these findings, we propose a set of concrete implications for institutions and nurse educators. First, institutions should provide protected time for nurses to engage in EBP activities, along with access to evidence resources within clinical workflows. Second, training programs should adopt blended learning designs that combine self‐directed online modules (as implemented in the #Evidencer Project) with synchronous tutoring sessions, peer learning communities, and mentoring by EBP champions. Third, nurse educators should differentiate instructional strategies according to the EBP level of the learner: foundational content (steps 0–2) may be effectively delivered online and independently, while advanced competencies (steps 3–6) require experiential, context‐embedded learning supported by the institution.

### 4.1. Study Limitations

As is inherent in qualitative research designs, the findings of the present study are not intended to be generalizable but may be transferable to similar clinical and educational contexts, particularly those involving experienced nurses engaging in voluntary, self‐directed EBP online training within national healthcare systems. Likewise, one of the limitations was that the expectations and experiences identified were closely related to the specific context and conditions in which the training took place. Lastly, to facilitate understanding and interpretation in other international contexts, it is important to note that all the Spanish nurses had a university degree, and nearly 40% also held a master’s degree, which may have influenced the results.

An additional limitation is the possible selection bias: participants had already collaborated in the previous phase of the project and had voluntarily completed the training, suggesting a prior level of motivation and interest in EBP above that of the general nurse population. Their experiences and assessments may not reflect those of professionals less engaged with EBP or who did not complete the course. It is also possible that other negative outcomes related to training were identified, in addition to those already mentioned. A further limitation relates to the potential interviewer effect (social desirability bias): despite the separation of roles between course designers and interviewers, participants may have been aware of the researchers’ involvement in the project, which could have influenced the nature of their accounts. This dynamic is an inherent feature of qualitative interviews and was minimized through reflective practice and consensus‐based analysis, as described in the section on reflexivity.

Data were collected through individual virtual semi‐structured interviews, which, while offering significant advantages in terms of geographical accessibility and scheduling flexibility, particularly relevant given the dispersed nature of the sample across hospital and primary care settings, may have limited the depth of nonverbal communication and the interpersonal rapport achievable in face‐to‐face settings.

## 5. Conclusions

This study analyzed the learning expectations and experiences of clinical nurses who participated in the #Evidencer Project, a self‐directed online training program on EBP. Three key findings emerged: first, the nurses’ motivation was primarily intrinsic and linked to professional development roles; second, the training was positively rated for its specificity, flexibility, and accessibility, although tutor support and downloadable materials were identified as areas for improvement; and third, the nurses’ understanding of EBP remains partial, focused on the early stages of the process, with persistent confusion between EBP and research. Based on these findings, we recommend that institutions regard training with the characteristics of the #Evidencer Project as one additional component within a broader strategy to improve EBP and implement changes in organizational culture through blended EBP training strategies that combine self‐directed online learning with tutored, context‐embedded practice, providing the organizational conditions: protected time, leadership support, and peer learning communities needed for the full implementation of EBP in clinical settings.

## Funding

This study was supported by a grant from the Spanish Ministry of Science and Innovation (Grant Number PID2019‐106545GA‐I00 funded by MCIN/AEI/10.13039/501100011033).

## Conflicts of Interest

The authors declare no conflicts of interest.

## Supporting Information

Additional supporting information can be found online in the Supporting Information section.

## Supporting information


**Supporting Information** This file includes illustrative direct quotes for each theme, subtheme, and codes. Table S1. Verbatim texts.

## Data Availability

The data are available from the corresponding author on request.
