# Supplementary material for: Clinical Nurses’ Expectations and Experiences of Self‐Directed Online Training in Evidence‐Based Practice: A Qualitative Study ‐ #EvidencerProject
Source: J Nurs Manag. 2026 Jul 3;2026:5549295. doi: 10.1155/jonm/5549295 (PMC13330135; doi:10.1155/jonm/5549295)
Supplement: Supplementary file 1 — Supporting Information This file includes illustrative direct quotes for each theme, subtheme, and codes. Table S1. Verbatim texts. [file JONM-2026-5549295-s001.docx]

Table S1. Verbatim texts

| **Theme** | **Subtheme** | **Code** | **Verbatim text** |
| --- | --- | --- | --- |
| Motivation and expectations about EBP training | Motivation | Interest in EBP | “I do little things every year, right now I don’t need it anymore, but I don’t stop training…the topic of evidence-based nursing is one of the things that most interests me, because I like being up to date” (E3: Doctorate).  “Well, I've already taken quite a few courses on evidence-based nursing. I'm getting older, and the more times I take a course on the same topic, the better I remember things.” (E7: Nursing).  Interest: “Well, above all, the issue of critical reading of articles was what interested me the most, which is where I was a little more, a little more green, that's mainly, and it has refreshed my knowledge” (E14: Specialty)  “I have found that it responded much more to needs that I had not detected or reinforced the part where I had weaker.” (E1: Doctorate). |
|  |  | Internalize and transmit to students | “Clarify concepts, … and then internalize them and be able to transmit them” (E1: Doctorate).  “We tutor Final Degree Project students, and thus, we want to provide quality” (E1: Doctorate).  “I tutor residents, so I am interested in going a little deeper into these topics.” (E14: Specialty). |
|  |  | Complement to other training | “It's just that since I was going to start the master's degree, I said, well, it will certainly be good for me to review a little and have the concepts clearer.” (E4: Nursing).  “I signed up to the course because I thought it could maybe serve me in the Research Master’s (which I’m currently doing)” (E2: Specialty).  “Well, I found it interesting, and I was just about to start the master's degree, I said, well, it would certainly be good for me to review a little and to have the concepts clearer, since I hadn't had much training before and then, because it seemed interesting to me.” (E4: Nursing)  “I was just starting my PhD program, so to learn a little more about research and above all, also at a more practical level, because the master's degree I did was more theory, and I think that's what I lacked, being able to apply it more.” (E5: Master’s) |
|  |  | Instrumental absence of EBP (there is no final motivation for doing something after this training) | No verbatim text |
|  | Expectations | Uncertain expectations with respect to online training | “No idea, I didn’t have any expectations, because I really didn’t know that I was signing up for” (E8: Nursing).  “I expected to have knowledge... to learn things because it's true that... that you know, you hear things, but no, you're not prepared for... for, for example, many bibliographic searches and many things that we still don't handle.” (E13: Master’s).  “I expected less than what it was…I expected the typical training , with some critical reading, or bibliographical search, which is what I often find at basic levels” (E11: Nursing)  “I admit that when I signed up I thought, maybe I'm signing up for something more like what I've done, right? (the professor) is going to explain four basic things to me. I admit I signed up with very little expectation, and then I was pleasantly surprised.” (E14: Specialty) |
| Experience with the #evidencer project’s online EBP training | Value of the training | “made to me” | “It has given me the feeling of being supported because, I say, it seems like we are present. It seems like someone has taken the time to teach us.” (E11: Nursing)  “It has examples from nursing, this is from nursing […] well I think they help us better when we see those examples, because sometimes in other courses they give you examples from medical studies and it seems you don't see how you can apply it in your daily practice..” (E5: Master’s).  “And I also think it's very practical because all the examples are from nursing research studies, so in our case, it kind of connects us more.” (E5: Master’s) |
|  |  | “A gift”: quality, clarity and free | “The free information…sometimes I have paid 1500 (euros) for it and it was disappointing…this is a gift” (E1: Doctorate).  “Useful, practical for your daily use, you can quickly access this training. Affordable, hasn't been a huge burden, and it's free" (E2: Specialty).  "The fact that the course is free is essential... the fact that it's free is very important."” (E6: Master’s). |
|  |  | “On my own” | “It seemed convenient to me” (E6: Master’s)  “It's labeled, so now you'll need a week or now you'll need two weeks or so. I've been able to organize myself” (E1: Doctorate).  “Well, that's the best thing about training because it gives you the opportunity to... when you have a moment to focus, you don't have to travel, you can... when you have a quiet moment at work, you enter (the course); when you're at home, relaxed, you go, that's for me... Great, because when you have to travel to a different place, in-person courses tend to be like you waste time when waiting for people to arrive or not. Here, for example, if you don't understand something, you just repeat it, you look it up again. That's what I like” (E9: Specialty)  “Well, in an in-person course, no, you don't understand everything well and you have to... you have to put up with it, but here it's different, you can stop for a while and then you can connect whenever you want, you take a little time, then you start with the course and that's what I like.” (E9: Specialty)  “Well, this course, as it is interactive, you can manage your own time. You can spend more hours. I, for example, spent more hours at night than during the day…I adapted it to my schedule… And then you advance at your own rhythm, you know? (E10: Master’s). |
|  |  | “I would recommend it to my colleagues.” | “Congratulations, because I loved it in fact, I have recommended it to some friends and they are also happy with it.” (E11: Nursing)  “Today I was telling one of my colleagues that it would have been good, that they could have done it, that maybe if there were more editions, that maybe it would have been good if they did it.” (E2: Specialty)  “I have a friend…and I also told her, sign up for this, it’s really good. It has an exceptional quality”. (E1: Doctorate) |
|  |  | Practical training | “Quite concise, practical and I really liked the explanatory videos.” (E14: Specialty).  "It gave me the feeling that they wanted us to learn key concepts, because they repeated them, or whatever they saw as being practical” (E4: Nursing).  “The course seemed quite practical and easy to me, because it is true that I have perhaps taken some other previous ones that have not been as practical for me” (E13: Master’s) |
|  | Usability of the training platform | Dynamic and attractive design of online training | “I think that, for being online, and the truth is that I have done quite a few courses, it was very dynamic, in blocks and it mixed videos with games, I don't know, it was entertaining and above all, the language seemed very direct to me, explaining things clearly without going around in circles, as if adding just the right amount of theory and practical.” (E4: Nursing)  “Then another thing is what we have discussed, very dynamic, with the presentations of Genially and all this and it seemed to me that it was great and then also the structure of the course.” (E5: Master’s)  "That’s why I really liked the platform, because it was very dynamic. Because it had videos, infographs..." (E2: Specialty) |
|  | Identification of improvements | Synchronous tutoring | “Having some tutoring like this to be able to express doubts directly to the teacher... some forum for doubts, some live (class)” (E5: Master’s)  “Well, a session with the professor so (he/she) can give us an explanation, well, especially for motivation, yes, for motivation” (E10: Master’s)  “I would have liked to the trainers in person more... well, maybe you could have explained it in a video, not so many puppets... it would have made it more relatable.” (E1: Doctorate). |
|  |  | Length and time spent | “Let’s see, I would add more (hours) with respect to the content, but yeah, I would leave it open longer, because the truth is that if you work, in the end you start to leave it, and it’s very hard to find time to sit down and do it." (E4: Nursing)  “Yes, I would add more hours by increasing the training a little more.” (E2: Specialty)  “The amount of time seems fine to me.” (E9: Specialty) |
|  |  | Downloading of materials | “Maybe if it had a paper copy… or a PDF where things are summarized, so I could consult it.” (E1: Doctorate).  “I would have liked to download some of the materials, because you forget it, and you cannot consult them” (E11: Nursing).  “The thing is, what I see as a disadvantage to the course is that I haven't been able to access the documentation that was available, which I found quite interesting and comprehensive. So there are times when, well, given the circumstances we each have, I'm one of those who saves all the documentation for the courses I take, the ones that seem interesting, to consult later and perhaps delve deeper into some topics, and that's what I've missed the most.” (E14: Specialty). |
| EBP Worldview | Global understanding of EBP | Complexity: reluctance, daily work | “They are difficult terms to understand, sometimes abstract, especially if no one explains them to you.” (E1: Doctorate).  “I need very little to try to change my practice because I'm so convinced, but... But I don't see in general in the unit, when a lot of people come through, a lot of contracts come in. I don't see that concern and I... and that's painful for me..” (E3: Doctorate).  “Yes, it’s an interesting subject. What happens is that I think of it as very complex…it is very difficult to…I think to internalize it into everyday life” (E8: Nursing)  “The reluctance of (male) colleagues… and (female) colleagues, nurses, and pediatricians, who may decide to do something because it's always been done that way, and it may be harder for them. No matter how much you provide, how much evidence you present.… (E1: Doctorate). |
|  |  | Provides critical thinking on clinical practice | “For me, a need to understand why I do things emerges” (E6: Master’s)  “When I started working... when I came back from working in all these places and started working here, because I started to think that the way we've always worked wasn't practical at all and it wasn't going anywhere.” (E7: Nursing)  “Well, I find it very interesting. Very important, almost essential, and... it started to interest me. I think even before I knew what it was, because I've always been the master of the whys: Why is this done this way? Why do we do this this way? This other thing... And then, once I saw what it was, I took courses, because I thought it was interesting to train on it because I have a degree. So, no, I didn't have much training on it during my degree because it was quite a few years ago. So, well, I'm delighted to continue training.” (E11: Nursing) |
|  | Training for my use, but not for patients |  | “Well... well, look, right now I'm working on a nutritional intervention project with a colleague. And it helps me... all this mental structuring to say this step after the other, because that directly affects my patients, both directly and indirectly. But of course, if I don't, I don't know how to search, I don't know how to decide what to read, and I don't know how to then express what... what I've learned, well, it's not going to benefit them either, I mean, more than direct attention to say in this specific situation. Well, it's a bit general, I think that... that what ultimately reaches them...” (E11: Nursing)  "They benefit from us doing things better, right? Then, maybe it is not a direct application towards them, but in the end, they benefit from what we do” (E8: Nursing)  “Well, in the end, they're the recipients of our care, so this has to move us. I mean, in the end, I don't like to say it like this, but the product is, in other words, the care that's for them, so to speak. So, we have to feel obligated to improve and learn all the time, for and by them.” (E2: Specialty)  “The role is to do everything for the good of the patient, and to do it for the good of the patient, we have to look for care that is truly supported, as if it will be beneficial for them.” (E5: Master’s)  "I don't think so. When it's generally taken into account, right? Because, well, there are professionals who do take it into account. In general, we're all overloaded with work, and no, we don't even have time to consider anything. And if the patient doesn't complain and doesn't impose themselves, it's ignored." (E7: Nursing) |
|  | EBP vs. research |  | “Because there are also times when we want to start a research project or something that the (hospital) floor, some case that was relevant, then to know how we could do it” (E13: Master’s)  “Well, I think the documentation is quite comprehensive. It's very complete, and it also helps you locate it and work on it, and it's like... because of the topic it covers, it seems like research is something we all have to deal with, and it seems more difficult, right? I found the way of acquiring knowledge quite accessible. Others show you two videos and you're already on the next level.” (E12: Master’s).  “Well, although I haven't been able to, maybe dedicate all the time I'd like to it... but quite a bit. Because I can't tell you about the first courses I started doing on research, and that was many years ago.” (E12: Master’s)  “Yes, look, as a nurse, and now that I know physical therapists… there's a great interest in research among physical therapists. They're much more inquisitive, they're much more inclined to go to conferences and do better… better quality work than us, than the nurses.” (E7: Nursing). |
|  | Individual vs. group | "I" as an independent person to learn  "I" as a professional in my clinical context | “I find it very interesting that there's a... like a little group on the (hospital) floor... man, it's very difficult for everyone to get on board, but the fact that there's a little group... because when you're a lone wolf, I've had that experience for many years and it's very, very difficult to do anything." (E3)  "It's always happened to me. I've always blamed my environment for being hostile, for not being comfortable, but as my life went on, I came to the conclusion that the problem was me. I came to accept that the problem was me, that people come into life to be happy, you have to be relaxed. So that makes me very sad. It's also that anything you set in motion costs a lot.".” (E6: Master’s)  "There are two people who are driving forces behind BPSO, because they spread the word to others or get them a little involved in things, so I do think it has a lot of influence.” (E4: Nursing)  “Yes, yes, right now I’m absolutely alone, the feeling…but the feeling is: stay in your consultation, do what you have to do, but don’t’ mess around” (E6: Master’s).  “We are working on it... because we want to do some clinical cases and things like... and so that's why I took this course, which several colleagues from the unit also took for the same reason, because it encouraged us to start” (E13: Master’s). |
